# On the connection between creativity and aesthetics

**DOI:** 10.3389/fpsyg.2024.1377485

**Published:** 2024-05-30

**Authors:** Steven Brown

**Affiliations:** Department of Psychology, Neuroscience & Behaviour, McMaster University, Hamilton, ON, Canada

**Keywords:** creativity, aesthetics, cultural evolution, variation, selection

## Abstract

Within cognitive psychology, there are separate experimental fields devoted to the study of creativity, on the one hand, and aesthetics, on the other, with virtually no cross-talk between them. In this article, I propose a means of uniting creativity and aesthetics via a consideration of the mechanisms of cultural evolution. I call this *the creativity/aesthetics cycle*. The basic tenet of the model is that creativity and aesthetics mediate, respectively, the processes of variation (production) and selection (perception or consumption) in evolutionary models of culture. By means of this cycle, creators produce works that they hope will be evaluated positively by consumers, where such appraisals ultimately feed back to influence the subsequent decision-making processes of creators. I discuss the implications of this model for the fields of creativity and aesthetics.

## 1 Creativity and aesthetics apart

Within cognitive psychology, there are separate experimental fields devoted to the study of creativity, on the one hand, and empirical aesthetics, on the other, with virtually no cross-talk between them (Tinio, 2013). Even an anthology with the tantalizing title of *Aesthetics and Innovation* (Dorfman et al., 2007) has chapters about either aesthetics or creativity, but not both. In this article, I present a proposal for uniting creativity and aesthetics through a model that I refer to as “the creativity/aesthetics cycle” (Brown, 2022). This perspective relies on an invocation of Darwinian models of cultural evolution. Such models provide a means of linking creativity and aesthetics in a recurrent manner. But let me first consider creativity and aesthetics as they are traditionally conceived in the experimental psychology literature.

The cognitive psychological study of creativity explores how people devise ideas or products that are considered to be novel and surprising (reviewed in the chapters of Kaufman and Sternberg, 2019). A dominant approach to the experimental study of creativity is based on the generative process known as divergent thinking (Guilford, 1967; Runco, 2010), which is a directed form of brainstorming. A popular psychometric test of this is the Alternate Uses task (Guilford, 1967; Torrance, 1974, 1988), in which people are asked to come up with as many unusual uses for a common object as possible in a 2-min time period. These uses are then rated by judges for their fluency, flexibility, originality, and elaboration, resulting in a metric of trait-level creativity (Runco, 2010; Plucker et al., 2019).

In contrast to creativity’s focus on production processes, the psychological study of aesthetics – hereafter referred to as *empirical aesthetics* to distinguish it from the philosophy of aesthetics – is oriented to perception, more specifically to the perceptual appraisal and experience of beauty in objects, including associated cognitive processes (Shiner, 2001; Chatterjee, 2014; Chatterjee and Vartanian, 2014; Pearce et al., 2016; Wassiliwizky and Menninghaus, 2021). The general field of aesthetics has its historical roots in the philosophy of perception from the 18th century (Crawford, 2001; Williams, 2009; Hayn-Leichsenring and Chatterjee, 2019), with a strong emphasis on the perception of visual art (Zeki, 1999; Leder et al., 2004). The field has a primary focus on *positive*-valenced aesthetic appraisals – mainly beauty and transcendence – to the exclusion of negative-valenced appraisals, although a number of theorists include negative-valenced emotions as aesthetic emotions, such as dislike (Zangwill, 1999; Saito, 2017), disgust (Rozin, 1999; Korsmeyer, 2011), terror (Berleant, 2009), and the negative sublime (Berleant, 2009). Aesthetics is, at its cognitive core, an emotional appraisal of the appeal of objects (Ortony et al., 1988), and so this applies comparably to negative and positive evaluations by perceivers. Finally, the field of empirical aesthetics gives no consideration to *where the appraised objects come from to begin with*. In other words, it lacks an account of the process of creation. It begins its analysis at the level of the finished product and how its perception impacts perceivers. For example, the interesting observation that viewers have a preference for curvilinear over rectilinear elements in architectural spaces (Vartanian et al., 2019) begs the question of how architects came to develop these features of building design to begin with. Outside of psychology, aesthetic philosophers make general reference to an artwork’s “history of production” as a non-aesthetic feature of the work (Zangwill, 1999), but they do so without referencing the processes by which creators imbue their products with aesthetic properties.

As will be discussed below, fields outside of cognitive psychology, such as design studies, *do* give consideration to how objects come to acquire their aesthetic features from creators (Crilly et al., 2004; Cropley and Kaufman, 2019; Goucher-Lambert et al., 2019; Han et al., 2021; Yang and Lu, 2022; Balters et al., 2023). However, I am going to argue that we need to distinguish two very different meanings of aesthetics in this field (Crilly et al., 2004; Wassiliwizky and Menninghaus, 2021): on the one hand, the intrinsic features of objects (i.e., their aesthetic properties, aesthetic appeal, or aesthetic value), and, on the other, the psychological responses of perceivers to these objects (i.e., aesthetic responses, aesthetic appreciation, or aesthetic experience). Design studies mainly emphasizes the former, namely how creators attempt to imbue their products with aesthetic value (Crilly et al., 2004). It views creativity (i.e., novelty) and aesthetics (i.e., appeal) as two key *dimensions* of product design. These dimensions may be co-present or co-absent in a creative product, depending on the specific object and the perceiver’s response to it. As Cropley and Kaufman (2019) point out, even within the field of design practice, industrial designers tend to be more oriented to aesthetics than are engineers, who tend to focus mainly on functionality. This jibes with the idea that some types of creativity are meant primarily to solve problems and provide functionality (e.g., vaccine design), whereas other types are more oriented towards display and attraction (e.g., fashion design).

Before delving into a proposed model of the relationship between creativity and aesthetics in psychology, I want to mention Tinio’s (2013) “mirror model” as an important cognitive precursor to the ideas developed here. This model relates the art-marking process of creators to the aesthetic experience of perceivers by means of inverted processing hierarchies in the two domains. More specifically, the model relates the progressive building up of an artwork by an artist – from the initial sketch to intermediate drafts to the final product – to a “reverse succession” for perception, going from early automatic processing, to memory-based processing, to high-level narrative interpretation and aesthetic processing. This correspondence results in the mirroring aspect of the model. Of course, the perceiver only ever sees the final work, and so the perceptual hierarchy that Tinio (2013) describes is only applied to the final work, and not the intermediate drafts.

## 2 Cultural evolution as a linkage

On the surface, creativity and aesthetics seem to be highly different from one another. So, how can creativity’s focus on novelty in production be united with empirical aesthetics’ focus on beauty in perception? I believe that a useful approach can be found in a consideration of Darwinian models of cultural evolution. The theory of cultural evolution adopts the fundamental Darwinian processes of variation and selection from biological evolution, but applies them to cultural objects and their transmission (Campbell, 1960; Cavalli-Sforza and Feldman, 1973; Boyd and Richerson, 1985; Durham, 1991; Mesoudi, 2011). Mechanisms for generating cultural variation lead to a diverse assortment of stylistic variants across all domains in a culture. The introduction of new variants does not, in and of itself, guarantee that these variants will become adopted as enduring components of a culture. Instead, there are social forces that allow certain variants (either old or new) to be transmitted to future generations and others to go extinct. This is conceptualized as a process of “cultural selection” (Boyd and Richerson, 1985; Durham, 1991; Mesoudi, 2011), analogous to natural selection.

## 3 Creativity and aesthetics combined

With this background about cultural evolution in mind, I want to make the claim that *creativity and aesthetics mediate, respectively, the processes of* var*iation (production) and selection (perception) in cultural evolutionary models*. I will refer to this relationship as the creativity/aesthetics cycle (Brown, 2022). I will present an analysis of this cycle that highlights a deep, but hitherto-undescribed, connection between creativity and aesthetics.

**Figure 1:**
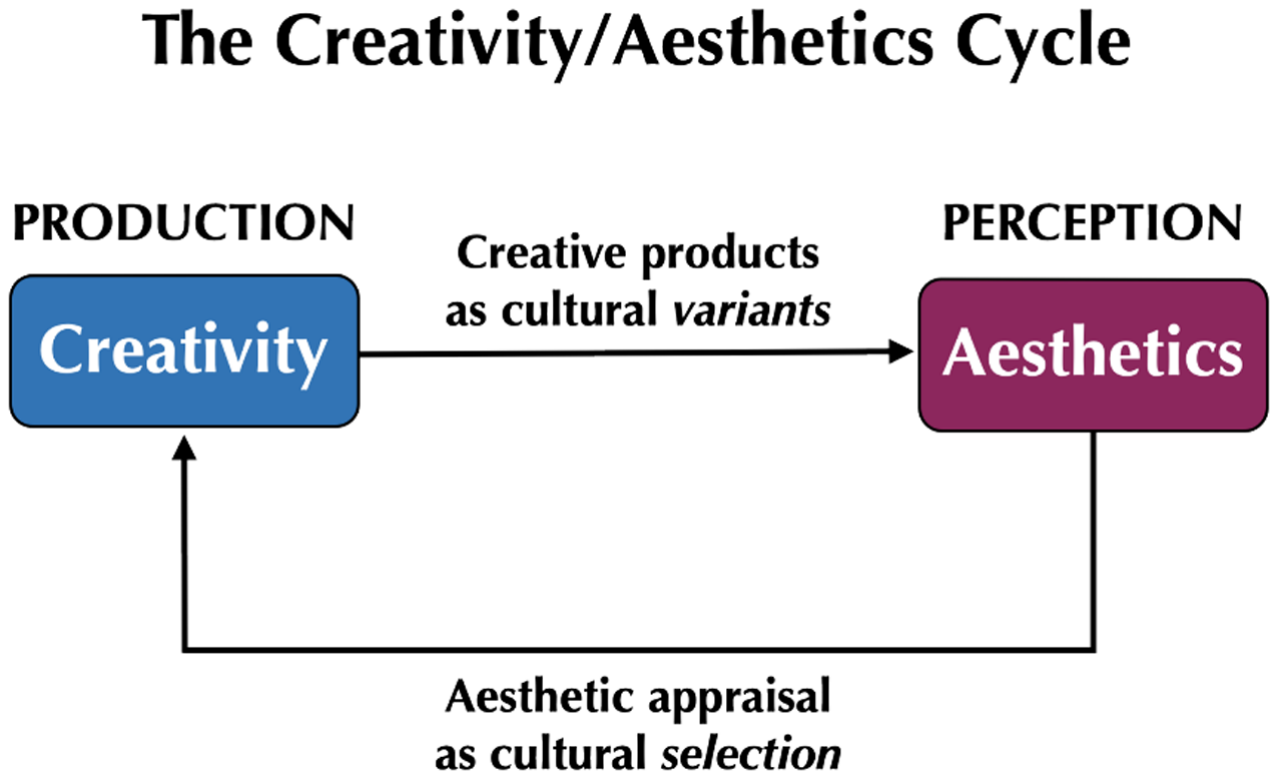
The creativity/aesthetics cycle. In cultural evolutionary models, creativity functions as a production mechanism that generates cultural variants, whereas aesthetics functions as an appraisal system to evaluate the products of creativity, thereby acting as a force of cultural selection. This motor/sensory loop is closed when the aesthetic appraisals of consumers influence the production choices of creators.

Figure 1 depicts the creativity/aesthetics cycle as a motor/sensory loop in which creativity is the production mechanism, and aesthetics is the perceptual mechanism. The motor/sensory loop is closed when the aesthetic appraisals of consumers feed back to modulate the decisions of creators and thereby influence the production of creative variants at their source. The proposal of a creativity/aesthetics cycle argues for a co-evolutionary relationship between creativity and aesthetics, similar to that for communication systems in which a production mechanism co-evolves with its evaluation mechanism (Prum, 2013).

### 3.1 Creativity as variation

Creativity is the production process in the model. Its output consists of creative products, which themselves comprise the variants in a model of cultural evolution. Creativity is the major generator of the diversity of stylistic variants in a culture (Csikszentmihalyi, 1988; Mesoudi, 2011; Fogarty et al., 2015; Gabora, 2018, 2019). Psychologically, the creative process involves the generation of ideas and their elaboration into finished products through the mediation of exploratory mechanisms and processes of revision (Finke et al., 1992; Mace and Ward, 2002; Mumford et al., 2012). Creators imbue their products with features that they hope will make them appealing to consumers of these products, whether this be directed to a global audience or a specialized subculture. In evolutionary analyses of the arts, Dissanayake (2009, 2018) refers to this process as “artification,” which is the mechanism by which people seek to make objects, whether constructed or natural, aesthetically appealing.

### 3.2 Aesthetics as selection

Aesthetics is the major mechanism for appraising the products of human creativity for their appeal, thereby influencing the likelihood that these products will get transmitted across generations. This provides a deep psychological linkage between creativity and aesthetics, a relationship that is rarely mentioned in the experimental literatures of either field. The creativity/aesthetics cycle serves as a motor/sensory loop between creativity as a production mechanism and aesthetics as a perceptual-evaluation mechanism linked to it. Because aesthetics is the appraisal system for the appeal of creative products, it is a major contributor to cultural selection. Hence, the aesthetic system serves as a kind of filter that allows certain variants to pass through to successive generations and others to go extinct.

In cultural evolution theory, this process of selection is mediated by a series of “transmission biases” that influence people’s preferences for some variants over others (Boyd and Richerson, 1985, 2005; Laland, 2004; Mesoudi, 2011), for example the bias to favor those products that are used by highly-esteemed individuals in a domain (Gil-White and Henrich, 2001). Conformity is perhaps the strongest force of cultural selection, such that people develop a preference for certain products because they are preferred by the majority of people (Boyd and Richerson, 1985; Sternberg and Lubart, 1995; Boyd and Henrich, 1998; Mesoudi and Lycett, 2009; Legare and Nielsen, 2015). The type of transmission bias that is perhaps most directly related to aesthetic appeal is the “content bias” (Rendell et al., 2011), in which people select objects based on their intrinsic features, including their aesthetic properties (among others). All of these selection biases operate by influencing people’s affinity for particular cultural variants. Therefore, the topic of selection biases in cultural evolutionary theory is intimately related to the psychology of aesthetics and decision making.

An alternative way of thinking about the creativity/aesthetics relationship outside of the experimental fields devoted to creativity and aesthetics alone comes from the fields of design studies and engineering. I mentioned the fact that empirical aesthetics is a perceiver-oriented domain that gives minimal consideration to where the aesthetic features of objects come from to begin. Design studies, by contrast, provides a more holistic perspective on the topic by examining how creators imbue their works with aesthetic features in order to make them appealing to consumers (i.e., artification). This encompasses what philosophers refer to as “everyday aesthetics” (e.g., Saito, 2017), in which aesthetics is examined beyond the traditional domains of fine art and nature.

**Figure 2:**
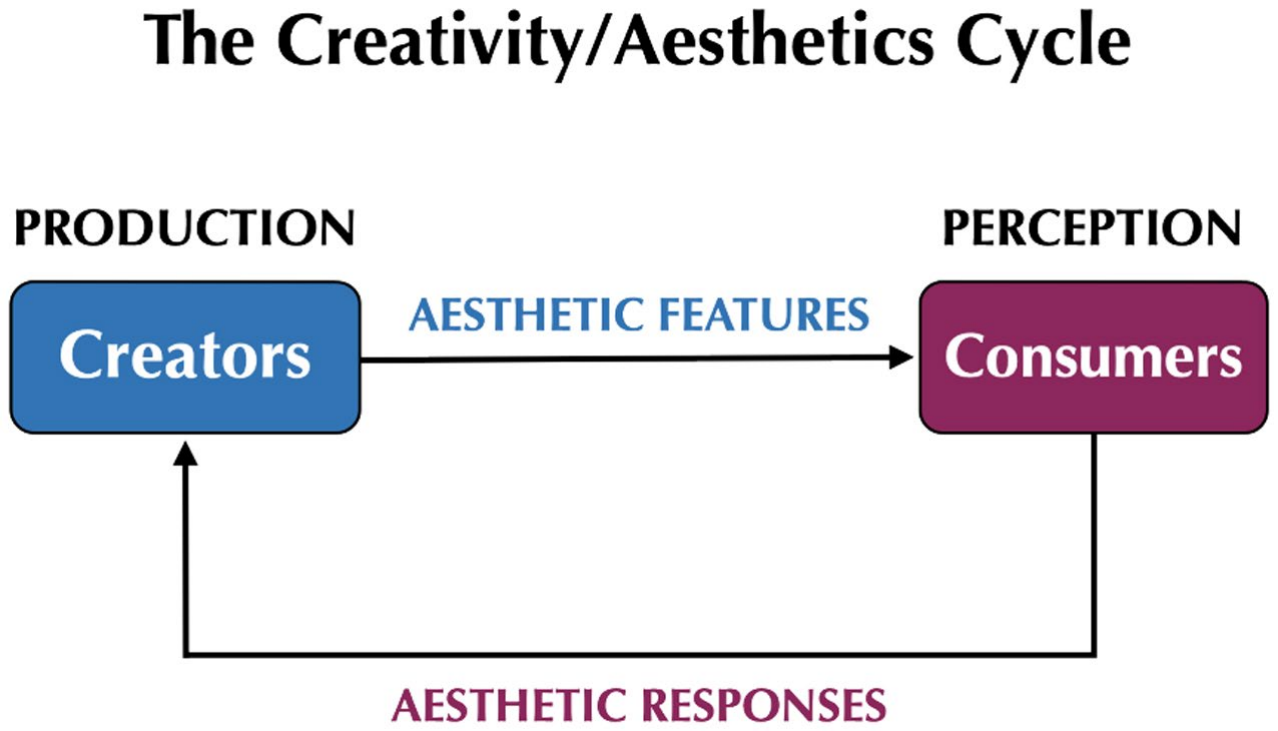
The creativity/aesthetics cycle reframed from the standpoint of the contrastive social roles of creators and consumers. Aesthetic features are an output of creators, whereas aesthetic responses are an output of consumers. Creators imbue products with aesthetic appeal – a process referred to as “artification” – whereas consumers select those products that they find aesthetically appealing. Note that novelty can be a type of aesthetic feature in a domain where novelty is considered appealing, such as technology.

The literature in design studies reveals that aesthetics is conceptualized in two different manners (Crilly et al., 2004; Han et al., 2021; Yang and Lu, 2022). On the one hand, it relates to *the features of an object* that people find attractive, in other words to an object’s aesthetic appeal or aesthetic value. This has connections with “formalist” approaches to philosophical aesthetics, which focus on the directly-perceivable properties of objects (see Kivy, 1980 for music, and Zangwill, 1999 for the arts more generally). On the other hand, it relates to *the psychological processes* in perceivers that are stimulated by their encounter with objects, namely their aesthetic evaluations, aesthetic responses, or aesthetic experience. Wassiliwizky and Menninghaus (2021) refer to this distinction as one between a stimulus-oriented and subject-oriented approach, respectively, to aesthetics. Along similar lines, aesthetic philosophers distinguish between the “property” of beauty and the “judgment” of beauty (Zangwill, 1999). This type of distinction is critical in thinking about the creativity/aesthetic cycle since *aesthetic features are an output of creators,* whereas *aesthetic responses are an output of consumers*, especially when such responses influence decision-making process related to consumption (see Figure 2).

The field of design studies mainly focuses on the objects themselves (“product aesthetics” in Crilly et al., 2004) and on how creators work to imbue their products with features that will be aesthetically appealing to consumers (i.e., artification). By contrast, the field of empirical aesthetics focuses on people’s aesthetic responses. While this is directly related to the features of perceived objects – indeed, these features generally comprise the independent variable in studies of empirical aesthetics – there is no connection to creators or the creative process. For example, a study that compares people’s evaluations of original paintings to those of forgeries is concerned with the viewers, not the creators (Rabb et al., 2018).

In order to accommodate this more social and pragmatic view of creativity, Figure 2 refashions the creativity/aesthetics cycle away from the cultural-evolutionary focus of Figure 1 toward a model of the contrastive social roles of creators and consumers. Creators imbue their products with features that they hope will be aesthetically appealing to consumers (Crilly et al., 2004; Cropley and Kaufman, 2019; Han et al., 2021). These are not the only features that creators hope will attract consumers; functionality and novelty are important as well. Consumers respond to these products and select the ones that they find aesthetically appealing, in addition to other features such as functionality, affordability, and novelty. This points to another interesting intersection between creativity and aesthetics, namely that *the novelty of a creative product has the potential to be aesthetically appealing to consumers* (Crilly et al., 2004), as in contemporary discussions of neophilia. At the same time, there is a definite tendency among both creators and consumers to view novelty and aesthetic value as competing dimensions of a creative product (Berg, 2014; Han et al., 2021), such that extreme novelty is not typically considered to be appealing.

### 3.3 Closing the loop

The capacity of aesthetic-appraisal processes to serve as a source of cultural selection effectively closes the motor/sensory loop of the creativity/aesthetics cycle. I mentioned earlier that the field of empirical aesthetics gives little consideration to where the appraised objects come from to begin with. Not only do they come from creators, but the aesthetic appraisals of consumers are ultimately directed toward creators. Critical reception by consumers has a strong modulatory effect on how creators approach their future projects. Importantly, despite the fact that the psychology of creativity examines how creators generate novel ideas and products, novelty may not be the principal design feature that attracts consumers to products. Instead, they may focus on properties such as functionality, aesthetic features, and/or affordability (Han et al., 2021). Novelty, however, may be an attractive feature for certain types of products, for example technological products or fashion.

Positive feedback increases the chances that a creator will produce works that are stylistically similar to those that were successful in the past, whereas negative feedback may force a creator to consider completely new directions in their work, among them imitating the products of more-successful creators (“success bias”; Acerbi, 2016). As Prum (2013) has noted in relation to musical composers, “the transformation of aesthetic judgments among observers dynamically [feeds] back upon the production of new musical compositions and performances by other composers and artists, and [fosters] the creation of additional aesthetic innovations in music” (p. 818). Critical failures may induce a creator to abandon their career, or a company to abandon a product. This is all the more so since there is very little room at the top of any given field. Most industries operate in a winner-take-all manner such that only a miniscule proportion of created products account for the vast majority of consumption in any given domain (Frank and Cook, 1996; Acerbi, 2016).

Looking at these issues in broader historical terms, contemporary conceptions of both creativity and aesthetics have common roots in Enlightenment thinking about the fine arts. Shiner’s (2001) book *The Invention of Art* provides a groundbreaking historical account of the conception of the artist and artwork from the ancient Greeks to the 20th century. The book reveals the parallel evolution of modern concepts related to creativity and aesthetics. The Enlightenment period saw the emergence of a new view of the artist as a “genius,” separate from the more practical-minded artisan. This view was based on freedom: “freedom from the imitation of traditional models (originality), freedom from the dictates of reason and rule (inspiration), freedom from restrictions on fantasy (imagination), freedom from the exact imitation of nature” (Shiner, 2001, p. 112). The same period saw the emergence of the concept of “the aesthetic” as a unique type of “refined or contemplative pleasure” (p. 6) that is induced by the perception of works of genius. The artwork’s function is to elicit a quasi-religious experience of beauty in the perceiver, rather than to have any utilitarian purpose. The aesthetic came to be associated with high-culture notions of taste, refinement, and ultimately class. Hence, our contemporary Western notions of creativity (as works of genius) and aesthetics (as emotional responses to works of genius) coevolved during the Enlightenment. This served to highlight not only the autonomy of the fine arts from other domains of human experience, but the distinction of artists from artisans, and elite individuals from the masses.

## 4 Creativity and aesthetics shared?

The creativity/aesthetics cycle conceptualizes the relationship between creativity and aesthetics as being a recurrent motor/sensory loop, as shown in Figures 1 and 2. However, might there also be a *sharing* of cognitive resources between creativity and aesthetics? In other words, might there be neural overlap between the production of novelty and the perception of beauty? If so, what type of process would underlie this sharing? Figure 2 suggests two possibilities for this, one in the creator and another in the consumer. The first is that the act of creation automatically triggers an aesthetic evaluation of one’s emerging creative product, especially in situations where a creator is actively striving to imbue their product with aesthetic value (i.e., artification). The second is that the perception of a creative product by a consumer automatically activates motor-planning areas involved in its production, by means of a mirror-type mechanism. Note that the latter scenario has similarities to Tinio’s (2013) mirror model, in which the act of perception might trigger covert processes of production (Sacheli et al., 2022).

Thus far, most analyses of the neural basis of creativity have been carried out separately from analyses of the neural basis of aesthetics. A number of activation likelihood estimation (ALE) meta-analyses of the functional neuroimaging literature have been performed individually for creativity (Gonen-Yaacovi et al., 2013; Boccia et al., 2015; Fogarty et al., 2015; Wu et al., 2015; Chen et al., 2020; Cogdell-Brooke et al., 2020; Brown and Kim, 2021) and aesthetics (Brown et al., 2011; Chuan-Peng et al., 2020). For divergent-thinking tasks like the Alternate Uses task, concordant areas of brain activation are found in the supramarginal gyrus, posterior cingulate cortex (part of the default mode network), and pre-supplementary motor area (pre-SMA). For motoric improvisation tasks like creative writing and musical improvisation, the results point to a role for motor-planning areas in these tasks, such as the pre-SMA and the dorsal and ventral regions of the inferior frontal gyrus. By contrast, aesthetic perception activates a very different set of brain areas, mainly limbic areas involved in emotional appraisal and reward, such as the anterior insula, ventral striatum, hippocampus, and orbitofrontal cortex.

Sacheli et al. (2022) carried out the first meta-analytic comparison between creativity and aesthetics, doing so in the domain of visual production/perception. As with the abovementioned ALE analyses, they performed separate ALE analyses for the creative production of drawings and the aesthetic perception of paintings. The results replicated previous analyses, showing the importance of premotor and sensorimotor areas for creative production, and limbic areas like the anterior insula and hippocampus for aesthetic perception. However, they also found evidence of a common area between creativity and aesthetics, namely the pre-SMA.

Of the two scenarios mentioned above, the observation of overlapping activity in the pre-SMA is more consistent with the engagement of motor-planning areas during aesthetic perception than of evaluative processing during creative production, although it could potentially be consistent with both models. The pre-SMA is a highly multifunctional area (Ruan et al., 2018), involved in all facets of executive functioning. Hence, it is an area that could potentially straddle motor planning and cognitive evaluation. Note that the aesthetic meta-analysis of Brown et al. (2011) did not report pre-SMA activation for the aesthetic processing of tastes and smells. Therefore, it is possible that the appearance of the pre-SMA for aesthetic processing in Sacheli et al.’s ALE analysis is a function of the visual modality used for aesthetic perception. It is more difficult to imagine a motoric counterpart to the aesthetic appraisal of tastes and smells. By contrast, the connection between vision and motor activity in the brain in found both in the “how” system for the visual guidance of hand movement (Goodale and Milner, 1992, 2018) and in the neural system for the visual guidance of eye movement (McDowell et al., 2008; Anderson et al., 2012).

Given that the results of Sacheli et al. (2022) are based on meta-analysis data alone, what is strongly needed is a neuroimaging study that examines creative production and aesthetic perception side by side in the same participants, rather than performing the comparison meta-analytically across literatures. This would provide the clearest means of knowing whether there are indeed shared processing areas for creativity and aesthetics, and whether the pre-SMA comes up as a significant brain area in a statistical conjunction analysis.

## 5 Conclusion

The experimental psychological fields devoted to creativity, on the one hand, and aesthetics, on the other, have tended to be completely separate domains, with their respective emphases on novelty (in production) and beauty (in perception). Design studies offers a more holistic view of things, although it tends to emphasize the creator and thus the properties of the designed product. I have proposed the creativity/aesthetics cycle as a means of uniting production (creativity) and perception (aesthetics) in the domain of cognition, as well as uniting variation (creativity) and selection (aesthetics) in Darwinian models of cultural evolution, and creators and consumers in cultural models of real-world creativity. The creativity/aesthetics cycle is a motor/sensory loop that is closed by the feedback of consumers on the products of creators. This process of cultural selection is reflected in the transmission biases posited by cultural evolutionists to account for the relative success of some variants over others. The creativity/aesthetics cycle provides a novel means of unifying two large fields in cognitive psychology that have historically had little to say about one another.

The implications of the model are widespread. These include the fact that all creators produce their work with the explicit goal of appealing to some target audience of consumers (i.e., artification), and that the aesthetic choices of consumers act as one of the strongest modulators of the decision-making processes of creators, at both the individual and organizational levels. Preliminary neuroimaging findings of a potentially shared brain area between creativity and aesthetics suggest that creative production might trigger an aesthetic evaluation of the generated product in the creator, and/or that aesthetic perception might trigger covert motor-production processes in the brains of consumers. Creativity and aesthetics are inextricably intertwined. This is so despite the fact that the experimental psychological fields devoted to them rarely make mention of their deep interconnections.

## Data availability statement

The original contributions presented in the study are included in the article/supplementary material, further inquiries can be directed to the corresponding author/s.

## Author contributions

SB: Writing – review & editing, Writing – original draft.
